# Consumer choice and preference heterogeneity for liquid milk attributes in Sri Lanka: evidence from a choice experiment

**DOI:** 10.3389/fnut.2026.1931191

**Published:** 2026-09-17

**Authors:** Sivakumar Sivashankar, Alan Renwick, Wei Yang, Krishnapillai Sooriyakumar

**Affiliations:** 1 Department of Global Value Chains and Trade, Faculty of Agribusiness and Commerce, Lincoln University, Lincoln, New Zealand; 2 Department of Agricultural Economics, Faculty of Agriculture, University of Jaffna, Jaffna, Sri Lanka; 3 School of Agriculture and Environment, Massey University, Palmerston North, New Zealand

**Keywords:** choice experiment, milk attributes, milk processing technology, random parameter logit model, willingness to pay

## Abstract

The majority of Sri Lankans rely on powdered milk over fresh liquid milk, despite liquid milk having a higher nutritional value than powdered milk. Also, the consumption of processed liquid milk remains low even among those who consume fresh milk. Therefore, understanding the key attributes that influence consumer choices is essential for developing effective policies and marketing strategies to promote the consumption of fresh liquid milk in Sri Lanka. This study uses a choice experiment approach to elicit consumer preference for liquid milk attributes from 410 consumers in the Northern Province of Sri Lanka. We used a random parameter logit model since consumers’ preferences are heterogeneous. This study focuses on five attributes of liquid milk—milk fat, processing technology, food safety measures, packaging, and price. The findings of this study reveal that respondents prefer low-fat milk, pasteurization and UHT processing technologies, enhanced food safety measures, and reusable packaging. The willingness to pay (WTP) estimates indicated that respondents were willing to pay an additional LKR 170.72 for low-fat milk compared with whole milk, LKR 99.13 for UHT-processed milk, and LKR 55.42 for pasteurized milk compared with milk without processing. Further, the results revealed that preferences for milk attributes are heterogeneous, indicating that respondents with different sociodemographic characteristics prefer different levels of attributes. The findings of this study will enable policymakers, marketers, and milk processing companies to understand the consumer preferences for liquid milk attributes, while providing information to develop products with varying attribute levels to meet consumers’ expectations and enhance competitiveness.

## 1 Introduction

The COVID-19 pandemic, followed by a severe economic crisis, has negatively affected the nutritional status of people in Sri Lanka (1). These crises caused disruptions in food supply chains and sharp increase in food prices, which reduced access to nutrient-rich foods and altered food consumption behavior (2). As highlighted by Weerasekara et al. (3), micronutrient malnutrition poses a serious threat to public health in Sri Lanka. Milk provides essential nutrients (4) and plays a vital role in supporting growth, health, and overall nutritional wellbeing (5). In the Sri Lankan context, promoting milk consumption is considered a key strategy to address nutritional deficiencies and improve overall dietary quality. Sri Lanka is one of the very few countries in the world that still consumes a large proportion of its milk in the form of powdered milk (6). Although both milk powder and processed liquid milk undergo heat treatment, liquid milk may retain higher levels of certain nutrients because milk powder production requires additional concentration and drying processes that may lead to nutrient losses. Milk powder production can result in losses of certain nutrients, such as lysine, vitamin A, tocopherols, riboflavin, and vitamin C (7). However, consumers in Sri Lanka tend to consume more powdered milk than fresh liquid milk. Notably, the consumption of fresh liquid milk in Sri Lanka is very low compared to other South Asian countries (8).

Although per capita dairy product intake has been gradually increasing in Sri Lanka, liquid milk consumption has been falling. According to the Department of Census and Statistics (DCS) of Sri Lanka, the average monthly per capita consumption of fresh liquid milk was 357.12 mL in 1990/91. However, this fell to 129.74 mL by 2019. In contrast, per capita milk powder consumption rose from 172.13 to 344.92 g over the same period. Before the advent of open economic policies in 1977, 80 percent of the country’s requirements were met by domestic milk production (9). However, since then, the import dependency has increased, and local milk production now satisfies only 45 percent of the domestic requirement, with the balance met through imports of milk products (10). Nevertheless, milk powder imports declined from 97,263 MT in 2020 to 49,526 MT in 2022, due to the impacts of the COVID-19 pandemic, global supply disruptions, foreign exchange shortages, and the economic crisis in Sri Lanka. However, imports showed a recovery trend from 2023 to 2025, increasing from 67,540 MT in 2023 to 79,471 MT in 2025.

Data from DCS indicate that, among liquid milk consumers, a significant proportion of Sri Lankan households consume unprocessed liquid milk over processed alternatives. In Sri Lanka, 56% of locally produced milk is marketed through the formal market, whereas the remaining 44% is distributed through the informal market (11), where milk is predominantly sold in unprocessed form. In the informal marketing channel, local producers sell raw milk directly to consumers without proper quality and safety standards, making it less reliable than imported powdered milk and processed liquid milk (6). Studies have shown that consuming unprocessed milk poses health risks due to the high risk of foodborne illness (12, 13). Meanwhile, processed liquid milk products, such as pasteurized, sterilized, and ultra-high temperature (UHT) milk, offer improved safety, longer shelf life, and convenience; however, their consumption among Sri Lankan consumers remains very low.

Liquid milk can have a range of attributes (full-fat, semi-skimmed, fortified, pasteurized, UHT, etc.). As a first step in boosting milk demand in Sri Lanka, we argue that it is important to understand which attributes are most valued by consumers. If manufacturers provide these preferred attributes, it could help increase demand, ultimately contributing to the country’s health and nutrition. In addition, replacing imported powdered milk with domestic fresh milk may benefit the Sri Lankan economy. However, no study has been conducted in Sri Lanka to examine consumer preferences for liquid milk attributes. Therefore, this study aims to fill the existing research gap and provide valuable insights into consumers’ preferences for various liquid milk attributes. Importantly, this study seeks to address two key research questions: What attributes do consumers value most in liquid milk? How do the sociodemographic characteristics of consumers influence their preferences for different milk attributes? Answering these questions has significant policy implications. This information may allow milk processing companies to allocate resources efficiently to produce liquid milk that meets consumers’ expectations. This is the first study investigating consumer preferences and willingness to pay for liquid milk attributes in Sri Lanka. This study’s findings may help businesses and milk processing companies to develop new products and improve existing ones.

## 2 Literature review

Consumers’ choices regarding liquid milk consumption are influenced by various milk attributes. Several studies have investigated consumers’ preferences for liquid milk attributes worldwide. Among the milk attributes, consumers’ preferences for milk fat content have been widely investigated by several researchers (14–18). Evidence from prior studies across various countries highlighted that milk fat is an important factor in the choices of liquid milk. The literature further shows that preferences for milk fat vary across countries.

In the literature, studies have investigated consumer preference for the heat treatment of liquid milk. Previous studies show that heat treatment and processing methods influence consumer choices in liquid milk consumption. Bai et al. (19), Sckokai et al. (18) assessed consumers’ preferences for the heat treatment attribute of liquid milk and reported a strong preference for pasteurized milk over UHT-treated milk in China and Italy, respectively. The literature indicates that nutrition claims have been a key determinant of milk purchases. For instance, Xu et al. (14) found that Chinese consumers preferred milk fortified with a combination of iron (Fe), calcium (Ca), zinc (Zn), and vitamin D over milk containing only vitamins A and D.

Food safety concerns have also been shown to play a vital role in determining consumer preferences for liquid milk. In addition, consumers’ trust in food certification and labeling, as well as in food chain actors, significantly shapes their purchasing behavior (20–22). For example, Sckokai et al. (18) found that Italian consumers were willing to pay for “reduced mycotoxins” milk, while Bai et al. (19) reported that Chinese consumers preferred milk with traceability labels, particularly those certified by the government or industry associations rather than third-party certification. Forbes-Brown et al. (23) revealed that Canadian consumers were willing to pay more for milk with a 100% Canadian label than for milk with no label. Further, Huang and Lee (24) found that Taiwanese consumers were willing to pay more for organic milk with an organic Certified Agricultural Standards (CAS) label, while Dhungel et al. (15) reported that Nepalese consumers were willing to pay more for liquid milk with a Good Manufacturing Practices (GMP) labeling attribute. These findings show that consumers are concerned about the food safety aspects of liquid milk when making purchasing decisions. Further, Jin et al. (25) reported that, when choosing liquid milk, Chinese consumers prioritized safety certification, shelf life, and nutritional value, while placing less importance on packaging and purchase location. The findings of earlier studies revealed that consumer preferences for certain liquid milk attributes vary across countries.

This study contributes to existing literature in several ways. First, several studies have been conducted worldwide to investigate consumer preferences for liquid milk attributes; however, no attention has been given to the Sri Lankan context, despite milk powder dominating the market. Second, this study contributes to the existing literature by providing evidence on how consumers’ sociodemographic characteristics influence their preferences for liquid milk attributes in Sri Lanka. Finally, the findings of this study will provide valuable guidance to milk processors, marketers, and policymakers in developing effective strategies and policies to boost liquid milk consumption in Sri Lanka.

## 3 Data and methods

### 3.1 Theoretical framework of choice experiment

The choice experiment is based on Lancaster’s consumer theory (26) and the random utility model of McFadden (27). Lancaster explained that the good itself does not provide utility for consumers; however, the characteristics of the particular good (i.e., attributes associated with that good) provide the utility (28, 29). For an individual *n* obtain utility (indirect) *U* from choosing alternative *i* from a finite set of *j* alternatives in choice task *C* in the *t* situation. The utility is modeled with a deterministic component (*V*_*nit*_)and a stochastic component (ε_*nit*_). Therefore, the utility (*U*_*nit*_) for the alternative *i* expressed as follows in Equation 1:


$$U_{n\,i\,t}=V_{n\,i\,t}+\varepsilon_{n\,i\,t}\;$$ (1)

$$U_{n\,i\,t}=V\,\left(X_{n\,i\,t},Z_{n}\right)+\varepsilon_{n\,i\,t}$$

The deterministic component (observable component) *V* is assumed to be a linear function of liquid milk attributes of an alternative *i* (*X*_*nit*_) and the sociodemographic characteristics of the respondent (*Z*_*n*_). *ε*_*nit*_ is the stochastic element (unobservable component). Hence, individual *n* will select alternative *i* if *U*_*nit*_ > *U*_*njt*_∀*j*≠*i*. Then, the probability of individual *n* choosing the *i^th^* alternative is expressed as follows in Equation 2:


$$P_{n\,i\,t}=\text{Prob}\left(V_{n\,i\,t}+\varepsilon_{n\,i\,t}>V_{n\,j\,t}+\varepsilon_{n\,j\,t}:\forall j\in C,\forall j\neq i\right)$$ (2)

$$=\text{Prob}\left(V_{n\,i\,t}-V_{n\,j\,t}>\varepsilon_{n\,i\,t}-\varepsilon_{n\,j\,t}:\forall j\in C,\forall j\neq i\right)$$

The Conditional logit model (CLM) can be used when ε_*nit*_ follows a Type I extreme value distribution assuming independently and identically distributed (IID), and the independence of irrelevant alternatives (IIA) (i.e., introducing another option does not influence the probability of choosing between the two alternatives) assumption is valid (30). The probability of *i^th^* alternative being chosen can then be expressed as in Equation 3:


$$P_{n\,i\,t}=\frac{e\,x\,p\,\left(V_{n\,i\,t}\right)}{\sum_{j}e\,x\,p\,\left(V_{n\,i\,t}\right)}$$ (3)

The CLM assumes that all respondents have the same preferences because all respondents share the same coefficients for all attributes. It will produce inaccurate results if tastes are heterogeneous across respondents (31). Among the alternative models to relax the limitations of the CLM (i.e., IIA), the random parameter logit model (RPL) is the most flexible and widely used within the literature on choice experiments. Also, the RPL model is a suitable approach for capturing consumer heterogeneity (30). The assumptions of independent and identical distribution (IID) and IIA are relaxed by the RPL model, and this permits the utility parameter to vary randomly among respondents (32–34). Following Train (35), the probability of respondents choosing the *i^th^* alternative is given as in Equation 4:


$$P_{n\,i\,t}=\int \frac{e\,x\,p\,\left(V_{n\,i\,t}\right)}{\sum_{j}e\,x\,p\,\left(V_{n\,i\,t}\right)}\,⨏\left(\beta |\theta \right)\,d\beta$$ (4)

Here, ⨏(*β*|θ) denotes the probability density function of parameter *β*. If the parameters are fixed at *β*_*c*_, the distribution collapses (30). We introduce an alternative-specific constant (ASC) into the deterministic component of utility. ASC corresponds to the *status quo* option, defined by dummy variables that take the value 1 for the *status quo* alternative and 0 otherwise. If there are *m* attributes, *V*_*nit*_ is expressed as in Equation 5:


$$V_{n\,i\,t}=\beta_{0}\,A\,S\,C+\sum \beta_{m}\,X_{m}$$ (5)

Where *β*_0_ is the coefficient of ASC, and *β* denotes the parameter of each attribute. The price attribute and ASC are assumed to be fixed, while other non-price attributes are considered as random and normally distributed parameters in the RPL model. Even though the RPL model accounts for preference heterogeneity by allowing the taste parameters to vary among respondents, it fails to explain the sources of preference heterogeneity (36).

To identify sources of heterogeneity in the RPL model, one can include interactions between respondents’ sociodemographic characteristics and the ASC and/or attributes (37). The definitions of the respondents’ specific variables employed to account for preference heterogeneity are given in Table 1. To avoid confounding with the grand mean, we used the effects coding^1^ (38).

**TABLE 1:** Respondents’ specific variables used to explain preference heterogeneity.

Variables	Description
Gender	Dummy variable 1 if the respondent is male, and 0 otherwise.
Age	Dummy variable 1 if the respondents’ age is above 40 and 0 otherwise.
Education	Dummy variable 1 if the respondent has completed a bachelor’s degree or above, and 0 otherwise.
Income	Dummy variable 1 if the household’s monthly income is above 50,000 LKR and 0 otherwise.
Household size	Dummy variable 1 if the household has four people or above and 0 otherwise.
Children	Dummy variable 1 if the household has children less than 18 years and 0 otherwise.

Willingness to pay (WTP) for each attribute is calculated by using the following Equation 6:


$$W\,T\,P_{k}=-2\,\frac{\beta_{k}}{\beta_{p\,r\,i\,c\,e}}$$ (6)

where WTP_k_ is the willingness-to-pay for the *k*th attribute, *β*_*k*_ denotes the coefficient for the *k*th attribute, and *β*_*price*_ is the coefficient for the price attribute. Since we used effect coding, the ratio between the attribute and price coefficients is multiplied by 2 (28, 31, 39). The confidence level intervals for the WTPs are estimated using the Krinsky and Robb (40) approach.

### 3.2 Attributes and levels

Selecting key milk attributes and appropriate levels is the most critical task in designing a discrete choice experiment (41). The attributes and levels used in this study (highlighted in Table 2) were selected based on expert opinion and focus-group discussions with consumers. In our choice experiment, we used five milk attributes: milk fat, processing technology, packaging, food safety measures, and price. The milk fat attribute varies by three levels: low-fat, non-fat, and whole milk. The processing technology attribute has four levels: pasteurization, sterilization, UHT, and no processing. The food safety measures attribute has two levels: an increase in food safety measures and no change. The price attribute contains five levels: LKR 150, LKR 200, LKR 250, LKR 350, and LKR 400. A pilot study involving 40 participants was conducted to obtain priors for an efficient design using Ngene software, and eight different choice sets were generated using a D-efficient design^2^. Each choice set consisted of three options: A, B, and C, with C representing the *status quo*^3^. Among these three options, respondents were asked to choose one option each time. An example of a choice set is shown in Supplementary file. Attributes were clearly explained to respondents before the choice experiment survey, and respondents who did not understand the questions were assisted by enumerators during the survey. We implemented an ex-ante method to mitigate hypothetical bias^4^ in our study. In each choice set, we reminded respondents to choose their true preferences and provided reminders for the opt-out alternatives.

**TABLE 2:** Summary of the attributes and levels used in this study.

Attributes	Levels
Milk fat	Low-fat
	Non-fat
	Whole milk (SQ)
Processing technology	Pasteurization
	Sterilization
	UHT
	No processing (SQ)
Food safety measures	Increase in food safety measures from current standards.
	No change (maintain current standards) (SQ)
Packaging	Single service packaging
	Reusable packaging (SQ)
Price	LKR 150 (SQ)
	LKR 200
	LKR 250
	LKR 350
	LKR 400

### 3.3 Study area and data collection

This study employed a multi-stage sampling technique, with the Northern Province purposively selected in the first stage and respondents randomly chosen from each district in the second stage. The questionnaire survey was conducted through face-to-face interviews in the Northern Province of Sri Lanka. The minimum sample size^5^ required for this study was 400. Individuals who were the primary shoppers and aged 18 years or older were invited to participate in the survey. The total number of questionnaires was distributed proportionally to the populations of the five districts in the Northern province. A total of 417 questionnaires were administered for the study. There were 410 questionnaires considered for final analysis after removing seven incomplete questionnaires.

## 4 Results

### 4.1 The respondents’ sociodemographic profiles

Table 3 compares the summary statistics of respondents’ sociodemographic characteristics with Sri Lankan Census data. Of the total respondents, 73.17% were female, and 26.83% were male. Our sample has a higher percentage of females than the 2019 Census, since females are the primary shoppers in most households. A total of 35.37% of respondents were between 18 and 29 years, 24.88% were between 30 and 39 years, 13.17% were between 40 and 49 years, and 18.05% were between 50 and 59 years. The smallest age group among respondents was 60 years or older (8.54%). Regarding the gender of the household head, the majority were male household heads (87.32%), and 12.68% were female. In terms of educational backgrounds of the respondents, 1.71% had only received their primary education, 4.88% had an education level between grade 6 to grade 10, 24.45% had passed their G.C.E. O/L, 48.90% had passed their G.C.E. A/L, 17.36% obtained a bachelor’s degree, and only 2.69% had a postgraduate qualification. Most respondents (45.00%) reported having a monthly household income of between LKR 25,001 and LKR 50,000. More than a quarter of respondents (27.37%) stated that their monthly income was less than LKR 25,000, whereas 16.32% reported monthly household income between LKR 50,001 and LKR 75,000, and 7.63% reported monthly household incomes between LKR 75,001 and LKR 100,000. A monthly household income of over LKR 100,000 was reported by 3.68% of respondents. The Census reports that the mean income in Sri Lanka was LKR 76,414^6^. Just under 32% had 1–3 members in their household, while 63.90% had 4–6, and only 4.15% had above six people. The Census reports that the mean household size was 3.7 persons. The Census reports that the mean household size was 3.7 persons. The survey results show that 48.31% of households had children under 18 years, and 51.69% did not have children under 18 years.

**TABLE 3:** Sociodemographic profiles of the surveyed respondents.

Variables	Group	Survey sample (%)	Census (%)
Gender	Male	26.83	47.20
	Female	73.17	52.80
Age (years)	18–29 years	35.37	–
	30–39 years	24.88	–
	40–49 years	13.17	–
	50–59 years	18.05	–
	Above 60 years	8.54	17.90
Gender of the household head	Male	87.32	74.70
	Female	12.68	25.30
Education	No schooling	0.00	3.10
	Up to grade 5	1.71	21.10
	Grade 6–10	4.88	44.00
	Passed G.C.E. (O/L)	24.45	16.70
	Passed G.C.E. (A/L)	48.90	12.20
	Bachelor’s degree	17.36	2.80
	Postgraduate	2.69	
Household monthly income (LKR)	Less than LKR 25,000	27.37	–
	LKR 25,001 to LKR 50,000	45.00	–
	LKR 50,001 to LKR 75,000	16.32	Mean of LKR 76,414
	LKR 75,001 to LKR 100,000	7.63	–
	Above LKR 100,000	3.68	–
Household size (persons)	1–3 persons	31.95	Mean of 3.7 persons
	4–6 persons	63.90	–
	Above 6 persons	4.15	–
Presence of children less than 18 years	Yes	48.31	–
	No	51.69	–

Source: Author’s calculations and The Department of Census and Statistics, Sri Lanka.

### 4.2 Econometric results

The model estimates and goodness-of-fit for the CLM and RPL models are reported in Table 4. Hausman and McFadden (42) test reveals that the IIA assumption is violated in the CLM and confirms that the CLM is not suitable for modeling the respondents’ preferences for liquid milk attributes in this study. Thus, we employed the RPL model for this investigation because it relaxes the IIA assumption^7^. We developed RPL models with interactions to identify the sources of heterogeneity among respondents in this study. Therefore, we estimated three RPL models with and without interactions: RPL-1 considers only main effects (i.e., ignores interaction terms); RPL-2 includes interactions between ASC and respondents’ sociodemographic characteristics; and RPL-3 includes interactions between milk attributes and respondents’ sociodemographic characteristics. The latent class model (LCM) was estimated for comparison and to check the robustness of the findings. Although both RPL and LCM approaches can account for preference heterogeneity, the extended RPL model was preferred because it allows the sources of preference heterogeneity to be examined by incorporating interactions between consumers’ sociodemographic characteristics and milk attributes. Also, the extended RPL model better aligns with the objective of examining how sociodemographic factors influence preferences for different milk attributes. The LCM results are provided in the Supplementary file.

**TABLE 4:** Estimation results of CL and random parameter logit (RPL) models.

Variable	CLM Coefficient	RPL-1 Coefficient	St. deviation	RPL-2 Coefficient	St. deviation
ASC	−0.598*** (0.092)	−0.841*** (0.107)	–	−1.130*** (0.179)	–
Price	−0.0038*** (0.0003)	−0.0055*** (0.0004)	–	−0.0055*** (0.0004)	–
Low-fat	0.295*** (0.041)	0.473*** (0.054)	0.359*** (0.073)	0.473*** (0.054)	0.359*** (0.073)
Non-fat	−0.080** (0.040)	−0.228*** (0.069)	0.936*** (0.068)	−0.226*** (0.069)	0.939*** (0.068)
Pasteurization	0.091** (0.046)	0.157*** (0.057)	0.018 (0.232)	0.153*** (0.057)	0.065 (0.266)
Sterilization	−0.025 (0.047)	−0.038 (0.065)	0.638*** (0.071)	−0.042 (0.065)	0.644*** (0.071)
UHT	0.187*** (0.040)	0.271*** (0.057)	0.524*** (0.072)	0.274*** (0.056)	0.483*** (0.075)
Food safety	0.143*** (0.023)	0.191*** (0.030)	0.223*** (0.049)	0.192*** (0.030)	0.222*** (0.049)
Packaging	−0.017 (0.021)	−0.051* (0.029)	0.228*** (0.044)	−0.051* (0.028)	0.211*** (0.046)
Interactions of respondents’ sociodemographic characteristics with ASC					
ASC × age				0.546*** (0.143)	
ASC × gender				−0.112 (0.159)	
ASC × education				−0.469** (0.194)	
ASC × income				−0.291* (0.173)	
ASC × children				0.151 (0.145)	
ASC × household size				0.251 (0.156)	
Log likelihood	−3320.29	−3060.78		−3044.44	–
AIC	6658.60	6153.60		6132.90	–
Sample size	410	410		410	–

***Denotes significance at 1% level.

**At 5% level.

* At 10% level. Standard errors are presented in parentheses.

All the models were estimated using maximum simulated likelihood procedures in NLOGIT software version 6.0, and the RPL model was estimated using 1,000 Halton draws. In all models, the ASC is statistically significant, and the negative sign indicates that moving away from the current situation may have a positive effect on the respondent’s utility. It suggests that, on average, respondents preferred selecting alternatives with improved milk attributes over the *status quo*. Moreover, the estimated standard deviations in the RPL model for all attributes except for pasteurization are significant. It reveals the existence of preference heterogeneity within the population.

#### 4.2.1 Estimation results of the random parameter logit model

The price coefficient is negative and significant at the 1% level, in line with economic theory, indicating that price increases reduce utility. The coefficient for the low-fat attribute is positive and statistically significant at the 1% level, and it has the largest coefficient among all evaluated attributes. The coefficient for the non-fat attribute is negative and significant at the 1% level. The coefficients for pasteurization and UHT are positive and statistically significant at the 1% levels. This indicates that consumers prefer liquid milk processed using pasteurization and UHT technologies. The coefficient for the food safety measures attribute is positive and significant at the 1% level, indicating that respondents would prefer an increase in food safety measures along the value chain of liquid milk from the existing food safety measures. Finally, the coefficient for the packaging attribute is negative and significant at the 10% level, indicating that respondents prefer reusable packaging over single-service packaging.

The results of the interactions between respondents’ sociodemographic characteristics and ASC are presented in Table 4. Our results show that respondents’ characteristics significantly influenced the choice of alternatives. The interaction terms between ASC and age, ASC and education, and ASC and income are significant, whereas the interaction between ASC and gender, ASC and children, and ASC and household size are not significant. The interaction term between ASC and age is positive and significant at the 1% level, revealing that, on average, respondents aged above 40 are more likely to choose the status quo alternative. In contrast, the interaction between ASC and education is negative and significant at the 5% level, indicating that, on average, respondents with higher levels of education are less likely to choose the status quo alternative. The interaction term between ASC and monthly household income is negative and significant at the 10% level, indicating that, on average, respondents with a monthly household income above LKR 50,000 are less likely to choose the status quo alternative.

#### 4.2.2 Results of the willingness to pay (WTP) estimation

The marginal WTP estimations are shown in Table 5. The WTP is calculated as the negative ratio between the attribute and price coefficient multiplied by two since effect coding was applied in this study. For this study, the confidence level intervals for the WTPs are estimated using the Krinsky and Robb approach with 1,000 draws in NLOGIT. We also calculated 95% confidence intervals for the WTP estimates using the delta method to verify the accuracy of the results obtained from the Krinsky and Robb (40) approach. The confidence intervals estimated using the delta method were very similar to those obtained using the Krinsky and Robb approach, confirming the robustness of the WTP estimates. The detailed results of the delta method are provided in the Supplementary file. In terms of milk fat, respondents are willing to pay LKR 170.72 more for low-fat milk and LKR 81.72 less for non-fat milk compared to whole milk. The estimated WTP for low-fat milk is higher than the WTP for other attributes, suggesting that low-fat is the attribute respondents value most. Also, our study revealed that respondents are willing to pay LKR 99.13 and LKR 55.42 more for UHT and pasteurization processing technology, respectively, compared to no processing. In addition, respondents are willing to pay LKR 69.50 for an increase in food safety measures for liquid milk above the current level. Moreover, respondents are willing to pay LKR 18.49 less for single-service packaging than reusable packaging.

**TABLE 5:** Estimated willingness to pay (WTP) for liquid milk attributes.

Variable	RPL without interactions	RPL with interactions
Low-fat	170.68*** [135.72, 205.63]	170.72*** [134.43, 207.00]
Non-fat	−82.29*** [−130.06, −34.52]	−81.72*** [−127.97, −35.48]
Pasteurization	56.79*** [16.82, 96.76]	55.42*** [15.23, 95.60]
Sterilization	−13.63 [−58.14, 30.88]	−14.99 [−62.68, 32.71]
UHT	97.66*** [57.79, 137.53]	99.13*** [60.84, 137.42]
Food safety	69.03*** [46.76, 91.30]	69.50*** [47.67, 91.33]
Packaging	−18.47* [−38.25, 1.32]	−18.49* [−38.86, 1.80]

***Denotes significance at 1% level.

*At 10% level. 95% confidence intervals are given parentheses.

The mean WTP values for the interaction effects between attributes and respondents’ sociodemographic variables are shown in Table 6. Respondents with a bachelor’s degree or higher qualifications are ready to pay LKR 73.26 more for low-fat milk than for whole milk, compared to those with lower educational qualifications. Respondents with larger household sizes are willing to pay LKR 92.01 less for low-fat milk compared to respondents with smaller household sizes. Individuals aged above 40 are willing to pay LKR 116.86 more for non-fat milk than those aged under 40. Households with children under 18 are willing to pay LKR 97.74 less for non-fat milk compared with households without children.

**TABLE 6:** Marginal willingness to pay (WTP) for interaction terms between attribute levels and respondents’ sociodemographic characteristics.

Variable	WTP
Low-fat × education	73.26* [−8.21, 154.74]
Low-fat × household size	−92.01*** [−163.17, 20.86]
Non-fat × age	116.86** [14.00, 219.72]
Non-fat × children	−97.74* [−199.94, 4.46]
UHT × income	86.20** [1.94, 170.47]
UHT × education	88.50* [−4.70, 181.71]
UHT × age	−116.00*** [−191.09, 40.91]
Packaging × gender	40.90* [−7.03, 88.83]
Packaging × age	−53.37** [−96.07, −10.66]
Food safety × education	65.84** [12.20, 119.48]
Food safety × age	38.71* [−0.76, 78.18]

***Denotes significance at 1% level.

**At 5% level.

*At 10% level. 95% confidence intervals are given parentheses.

Respondents possessing monthly household incomes greater than LKR 75,000 are willing to pay an additional LKR 86.20 on UHT processing technology compared to their counterparts. In addition, respondents with a bachelor’s degree or higher qualifications have shown a willingness to pay LKR 88.50 more for UHT processing technology compared to their counterparts. Consumers above 40 years are willing to pay LKR 116.00 less than those below 40 years for UHT processing technology. Male respondents exhibited a willingness to pay LKR 40.90 more than female respondents for single-service packaging. Further, older respondents are willing to pay LKR 53.37 less for single-service packaging than younger respondents. Respondents with a tertiary educational qualification and those aged above 40, are willing to pay LKR 65.84 and LKR 38.71 more, respectively, for enhanced food safety measures from milk collection to processing, compared to their counterparts.

## 5 Discussion

This study investigated Sri Lankan consumers’ preferences for liquid milk attributes and the influence of sociodemographic characteristics on their preferences. The demographic characteristics of the survey respondents differed from those reported in the Sri Lankan Census, particularly with respect to gender. This difference is mainly due to the survey design, which targeted the primary household shopper responsible for milk and food purchases. In many Sri Lankan households, food purchasing decisions are more frequently made by women, resulting in a higher proportion of female respondents in the sample. In this study, respondents’ socio-demographic characteristics, including age, education, and income, significantly influenced their choice of alternatives in this study. We found that young, tertiary-educated, and higher-income consumers prefer liquid milk with improved attributes compared to the currently available unprocessed milk. The reason may be individuals with higher levels of education are better aware of the benefits of improved milk attributes over currently available raw milk attributes. This finding is consistent with Habiyaremye et al. (43), who reported that consumers with formal education demonstrated greater awareness of milk safety issues, including the presence of bacteria and pathogens in raw milk, and showed a stronger preference for high-quality milk.

Our study found that Sri Lankan consumers showed a stronger preference for low-fat milk, reflecting growing health consciousness. This finding is consistent with Xu et al. (14), Harwood and Drake (17), who found that consumers in China and the USA, respectively, prefer liquid milk with low-fat content; however, it contrasts with the findings of Bekele et al. (16), Sckokai et al. (18), Dhungel et al. (15), who found that consumers in Ethiopia, Italy and Nepal, respectively, prefer high-fat milk. This difference in consumers’ preferences may be due to variations in dietary habits and nutritional perceptions regarding milk fat across countries (44). Further, tertiary-educated consumers are willing to pay more for low-fat milk than for whole milk, indicating greater concern about milk fat content. However, households with larger family sizes exhibit a negative willingness to pay for low-fat milk. In the market, low-fat milk is priced higher than whole milk, leading to higher food expenses for households with more members when they purchase higher-priced options. This could explain why households with more members are less willing to pay for healthier choices (45).

Our results showed that consumers had a negative preference for non-fat milk over whole milk. This finding contrasts with those reported by Rajo et al. (46), who found that consumers in the USA prefer skimmed milk to whole milk. It highlights that Sri Lankan consumers accept reduced fat content; however, they show a negative preference for the complete removal of fat content of liquid milk. It clearly shows that their preferences are influenced not only by health considerations but also by taste and other sensory qualities. Interestingly, we found that older consumers are willing to pay for non-fat milk, suggesting that people pay more attention to milk fat as they age. This finding is supported by Alsubhi et al. (44), who reported that elderly people are willing to pay more for healthier food. In contrast, households with children showed a negative preference for non-fat milk, suggesting that parents with children in the household believe that whole milk is more essential than non-fat milk for their children’s growth and development. Our finding is supported by Vanderhout et al. (47), who denoted that parents perceive whole milk as providing better nutrition for their children than reduced-fat milk.

Furthermore, Sri Lankan consumers preferred heat-treatment technologies such as pasteurization and UHT, whereas sterilization techniques did not significantly influence their preferences. This suggests that consumers may perceive the advantages associated with processing of liquid milk, such as safety, longer shelf life, and the convenience of pasteurized and UHT milk. Our results exhibited that household income and education strongly influence preferences for processed milk in Sri Lanka. Although UHT milk is more expensive than unprocessed milk in the Sri Lankan market, higher-income households are more willing to pay for it due to safety and convenience benefits. Educated consumers are more likely to acquire information on the safety aspects of liquid milk processing and, therefore, are willing to pay more for UHT processing technology. This is supported by Nam and Suk (45), who reported that literate consumers better understand the nutritional and health benefits of foods. Meanwhile, older consumers exhibited a negative preference for UHT milk; this may be due to their unfamiliarity with the processing technologies used in liquid milk. This finding is consistent with Li et al. (48), who reported that older consumers are less likely to pay for new food attributes.

Furthermore, the results showed that consumers are willing to pay for enhanced food safety in the milk value chain. This clearly shows that consumers are concerned about the safety practices across the entire milk value chain. This finding aligns with Habiyaremye et al. (43), who found that consumers in Rwanda are willing to pay more for milk when it is provided with milk safety and quality information. Our findings reveal that respondents’ education and age influence their willingness to pay for enhanced food safety measures in the milk value chain. Respondents with higher educational attainment may have greater awareness of food safety risks along the value chain and are therefore more likely to be willing to pay for improvements in food safety. These findings are supported by Can (49), Ali and Ali (50), Joya et al. (51), who denoted that individuals with higher educational attainment have shown a greater willingness to pay for food safety attributes. Moreover, older consumers are more health-conscious than younger consumers, making them more willing to pay for improvements in food safety. This finding aligns with Habiyaremye et al. (43), who found that elderly consumers in Rwanda were willing to pay more for safe and quality milk than younger consumers; however, it contrasts with the findings of Cheng et al. (52), who revealed that individuals over 60 years old are less willing to pay for milk safety in urban China.

The positive preference for reusable packaging indicates a growing awareness of the environmental impacts of packaging materials and a willingness to support sustainable practices. Neill and Williams (53) found that consumers in Texas prefer milk packaged in a returnable glass container. In contrast, Dhungel et al. (15) reported that Nepalese consumers preferred paper jar packaging over plastic pouches and exhibited a negative preference for paperboard compared to plastic pouch packaging. Our findings revealed that males and those aged under 40 exhibit a higher willingness to pay for single-service packaging than for reusable packaging, suggesting that these consumer groups prioritize convenience.

This study has several limitations. Due to time and financial constraints, the survey was conducted only in the Northern Province of Sri Lanka. The survey sample was not fully representative of the Sri Lankan population. Compared with national census data, the sample included a higher proportion of female respondents and differed in some socio-demographic characteristics, such as education. Therefore, the findings may not fully represent consumers’ preferences across the country. In this study, the survey primarily targeted household shoppers, who were predominantly female; therefore, the findings may not fully capture the preferences of the wider consumer population. Future studies should include respondents from all provinces of Sri Lanka and a more diverse sample of consumers to improve the representativeness of the findings and better reflect the preferences of the overall population. In addition, this study considered only five liquid milk attributes: milk fat content, processing technology, food safety measures, packaging, and price. Thus, future studies could consider other attributes of liquid milk to better understand consumers’ preferences. Although an ex-ante approach was applied to reduce hypothetical bias in this study, respondents evaluated hypothetical choice scenarios rather than making actual market purchases, which may lead to differences between estimated and real-world willingness to pay. Despite these limitations, this study provides valuable insights into consumer preferences and WTP for liquid milk attributes among the surveyed respondents.

## 6 Conclusion

This study provides new insights into consumer preferences for liquid milk attributes in Sri Lanka, where milk powder consumption remains significantly higher than fresh liquid milk. The study found that respondents prefer low-fat milk, pasteurization and UHT processing, improved food safety, and reusable packaging, and are willing to pay more for these attributes. The findings provide valuable insights for milk processors and marketers, who should diversify liquid milk products, as preference heterogeneity was observed among consumers in Sri Lanka. The findings of this study highlight that different respondents have different priorities regarding liquid milk; therefore, marketers and milk processors should produce liquid milk with varying levels of milk attributes to meet consumer expectations and increase the competitiveness of their products. Therefore, delivering liquid milk with consumers’ preferred attributes may increase consumer satisfaction and motivate consumers to select liquid milk that matches their desired attribute levels.

## 7 Policy implications

In Sri Lanka, only a few milk processing companies are involved in processed liquid milk production. Therefore, there could be a case for the government to encourage new entrants into the market, provide incentives for investment in dairy processing, and support smaller producers to help them enter the market. It will increase competition among domestic milk processors and lead to a greater diversity of liquid milk products to meet consumers’ preferences and dietary needs. In addition, encouraging new entrants into the market may introduce new technologies, increase supply, and create employment opportunities. The findings revealed that consumers are willing to pay for an increase in food safety measures compared to currently existing food safety measures; therefore, milk producers and marketers should implement appropriate food safety measures at each stage of the liquid milk value chain. Also, the government should promote good agricultural practices and ensure proper hygiene and safety standards during milk collection, processing, packaging, transportation and storage. Finally, promoting the consumption of fresh liquid milk not only improves human health and prevents nutritional deficiencies among the population, but also contributes to the growth of the domestic liquid milk industry in Sri Lanka. Therefore, policymakers and milk processing companies may develop suitable programs, such as educational campaigns and promotional activities, to increase awareness regarding the health and nutritional benefits of consuming fresh liquid milk.

## Data Availability

The raw data supporting the conclusions of this article will be made available by the authors, without undue reservation.

## Footnotes

1 Effects coding is an alternative to dummy coding, and effects coding creates L-1 variables similarly to dummy coding. In the effects coding, the level (non-reference level) of the qualitative variable takes 1 when that level is present and 0 otherwise and takes a value of −1 when the reference level is present (54).

2 Detailed choice experiment design is available in the Supplementary file.

3 We include a *status quo* (SQ) option in each choice set to make choices more realistic, and it helps not force respondents to choose liquid milk with different combinations of attributes that are not satisfactory to them. The status quo option refers to currently available liquid milk that price LKR 150 and has the following attribute levels: whole milk, no processing, reusable packaging, and no change in food safety measures. The attribute levels which are tied to the status quo option are considered as the base categories.

4 Hypothetical bias is defined as the difference between the amount of money people state they are willing to pay in hypothetical experiments and what they would actually pay in real purchase situations (55).

5 Following Wakjira and Kefale (56), the minimum sample size was calculated by the following equation: $n=\frac{N}{1+N\,\left(e^{2}\right)}$, Where, *n* = number of respondents, *N* = population size, and e = sampling error/level of precision.

6 Based on the HIES-2019 by the Department of Census and Statistics, Sri Lanka, households are classified into three income groups: low-income (average monthly income: LKR 17,572), middle-income (average monthly income: LKR 56,079), and high-income (average monthly income: LKR 196,289).

7 Also, the CLM and RPL models are compared using a likelihood ratio test to determine which model best fits the data. The value of the likelihood ratio test is 519.03, with a critical value of $\chi_{7\,\mathrm{df}}^{2}$ 0.815 at a significance level of 0.05. The likelihood ratio test rejects the null hypothesis that the CLM fits the data better than the RPL model. Hence, the RPL model is more suitable than CLM in this study to estimate the respondents’ preferences for liquid milk attributes.
