# Supplementary material for: Consumer choice and preference heterogeneity for liquid milk attributes in Sri Lanka: evidence from a choice experiment
Source: Front Nutr. 2026 Sep 17;13:1931191. doi: 10.3389/fnut.2026.1931191 (PMC13626999; doi:10.3389/fnut.2026.1931191)
Supplement: Supplementary file 1 [file Table_1.docx]

**Choice experimental design**

There are various methods for creating experimental designs, but efficient designs are currently the most popular. A full factorial design includes every possible combination of attribute levels. Mathematically, a full factorial design will generate $\prod_{k=1}^{K} L_{k}$ choice situations. Here, $L_{k}$ is the number of levels associated with the corresponding attribute k. A design with five attributes- two attributes with two levels, one with three levels, one with four levels, and one with five levels will generate 2×2×3×4×5=240 choice situations. Presenting many choice situations may place a significant level of cognitive burden on respondents. Therefore, a D-efficient design was employed to maximize information from each choice situation and increase the precision of parameter estimates. The D-efficient experimental design is the most commonly used in constructing experimental designs because it has smaller standard errors than orthogonal designs. Prior parameters are important when creating efficient designs, while choosing the wrong priors may lead to statistically inefficient designs. Therefore, we conducted a pilot study to obtain prior parameters for attributes. Priors can also be obtained from the literature in the absence of a pilot study. A pilot study with 40 participants was conducted to determine the priors for an efficient design. The Ngene software was used to create an efficient design with priors from a pilot study, and eight different choice sets were generated using a D-efficient design. The final survey was developed with a single choice block; therefore, all respondents received the same set of choice tasks.

Example of choice set.

Please select the alternative that you most prefer

| **Attributes** | **Alternative A** | **Alternative B** | **Status quo** |
| --- | --- | --- | --- |
| Milk fat | Whole milk | Non-fat milk | Whole milk |
| Processing technology | Pasteurization | UHT | No processing |
| Food safety | No change | Increase | No change |
| Packaging | Single service packaging | Reusable packaging | Reusable packaging |
| Price (per litre) | LKR 400 | LKR 250 | LKR 150 |
| **I choose** |  |  |  |

Table 1. Estimated WTP for liquid milk attributes using the delta method

**Variable	RPL without interactions	RPL with interactions**

Low-fat	170.68***	[134.32, 207.03]	170.72***	[134.34, 207.10]

Non-fat	-82.29***	[-130.10, -34.48]	-81.72***	[-129.78, -33.66]

Pasteurization	56.79***	[17.11, 96.48]	55.42***	[15.61, 95.22]

Sterilization	-13.63	[-60.09, 32.83]	-14.99	[-61.76, 31.79]

UHT	97.66***	[58.01, 137.32]	99.13***	[60.05, 138.20]

Food safety	69.03***	[46.44, 91.63]	69.50***	[46.92, 92.07]

Packaging	-18.47*	[-38.59, 1.66]	-18.49*	[-38.40, 1.41]

**Interactions of attribute levels with respondents' sociodemographic characteristics**

Low-fat × Education	-	73.26*	[-8.20, 154.72]

Low-fat × Household size	-	-92.01***	[-160.29, -23.74]

Non-fat × Age	-	116.86**	[18.93, 214.79]

Non-fat × Children	-	-97.74*	[-197.28, 1.78]

UHT × Age	-	-116.00***	[-189.77, -42.22]

UHT × Education	-	88.50*	[-4.19, 181.20]

UHT × Income	-	86.20**	[-7.93, 89.73]

Food safety × Age	-	38.71*	[-2.03, 79.45]

Food safety × Education	-	65.84**	[12.48, 119.20]

Packaging × Age	-	-53.37**	[-95.73, -11.00]

Packaging × Gender	-	40.90	[-7.93, 89.73]

*** denotes significance at 1%, ** at 5%, * at 10% level; 95% confidence intervals are given parentheses

**Latent Class Model**

Table 2. Criteria for determining the number of latent classes.

**Number of	Number of	Log likelihood	AIC	BIC**

**classes	parameters (P)	(LL)**

1	8	-3325.78	6667.56	3349.85

2	21	-3120.00	6281.80	3183.17

3	34	-2994.35	6056.70	3096.62

**4**	47	-2934.57	5960.80	3074.78

5	60	-2906.24	5932.48	3086.73

6	73	-2898.92	5943.84	3118.51

AIC (Akaike Information Criterion) is calculated as follows: -2(LL-P).

BIC (Bayesian Information Criterion) is calculated as follows: -LL+[(P/2) * ln(N)]

Table 3. Estimates of Latent Class Model

**Variable	Class 1	Class 2	Class 3	Class 4**

ASC	2.679***	1.235***	-0.910***	2.075***

(0.930)	(0.317)	(0.316)	(0.541)

Price	0.0011	-0.0081***	-0.0057***	-0.002

(0.001)	(0.001)	(0.001)	(0.002)

Low-fat	0.338***	0.410***	0.667***	-0.043

(0.110)	(0.081)	(0.146)	(0.191)

Non-fat	0.477***	0.015	-0.322*	-1.825***

(0.157)	(0.100)	(0.178)	(0.239)

Pasteurization	0.088	0.001	0.278*	-0.102

(0.120)	(0.089)	(0.145)	(0.221)

UHT	0.119	0.291***	-0.201	0.6423**

(0.117)	(0.085)	(0.152)	(0.256)

Food safety	0.266***	0.061	0.430***	0.467***

(0.064)	(0.088)	(0.084)	(0.162)

Packaging	0.035	-0.072	-0.099	0.174

(0.054)	(0.046)	(0.097)	(0.154)

Class membership estimates

Age	1.508***	0.905	1.662***	-

(0.574)	(0.574)	(0.638)

Gender	-0.198	-1.275**	-0.693	-

(0.613)	(0.564)	(0.621)

Education	0.200	-0.827*	-0.612	-

(0.471)	(0.485)	(0.559)

Income	-0.238	-1.620**	-1.482*	-

(0.630)	(0.669)	(0.866)

Constant	0.515	3.625***	1.578	-

(1.229)	(1.095)	(1.389)

Average class probabilities	0.231	0.430	0.217	0.122

Log likelihood function	-2933.40

Pseudo R2	0.186

*** denotes significance at 1%, ** at 5%, * at 10% level. Standard errors are presented in parentheses.

Table 4. Estimated WTP

**Variable	Class 1	Class 2	Class 3	Class 4**

Low-fat	-	101.37***	234.42***	-

[51.89, 150.86]	[116.92, 351.92]

Non-fat	-	3.71	-113.38*	-

[-44.16, 51.59]	[-230.72, 3.97]

Pasteurization	-	0.35	97.63*	-

[-42.65, 43.35]	[3.69, 191.58]

UHT	-	72.04***	-70.50	-

[32.31, 111.76]	[-173.90, 32.89]

Food safety	-	15.05	150.88***	-

[-31.45, 61.56]	[62.11, 239.65]

Packaging	-	-17.70	-34.79	-

[-39.74, 4.35]	[-104.10, 34.51]

*** denotes significance at 1%, ** at 5%, * at 10% level; 95% confidence intervals are given parentheses
